# Cervical cancer cell lines expressing NKG2D-ligands are able to down-modulate the NKG2D receptor on NKL cells with functional implications

**DOI:** 10.1186/1471-2172-13-7

**Published:** 2012-02-08

**Authors:** Miriam I Jimenez-Perez, Luis F Jave-Suarez, Pablo C Ortiz-Lazareno, Alejandro Bravo-Cuellar, Oscar Gonzalez-Ramella, Adriana Aguilar-Lemarroy, Georgina Hernandez-Flores, Ana L Pereira-Suarez, Adrian Daneri-Navarro, Susana del Toro-Arreola

**Affiliations:** 1 Laboratorio de Inmunología, Departamento de Fisiología, Centro Universitario de Ciencias de la Salud, Universidad de Guadalajara, Guadalajara, Jalisco, Mexico; 2 División de Inmunología, Centro de Investigación Biomédica de Occidente, Instituto Mexicano del Seguro Social, Guadalajara, Jalisco, Mexico; 3 Departamento de Ciencias de la Salud, Centro Universitario de los Altos, Universidad de Guadalajara, Guadalajara, Jalisco, Mexico

**Keywords:** NK cells, NKG2D, MICA, MICB, ULBP, Cervical cancer

## Abstract

**Background:**

Cervical cancer represents the third most commonly diagnosed cancer and the fourth leading cause of cancer-related deaths in women worldwide. Natural killer (NK) cells play an important role in the defense against viruses, intracellular bacteria and tumors. NKG2D, an activating receptor on NK cells, recognizes MHC class I chain-related molecules, such as MICA/B and members of the ULBP/RAET1 family. Tumor-derived soluble NKG2D-ligands have been shown to down-modulate the expression of NKG2D on NK cells. In addition to the down-modulation induced by soluble NKG2D-ligands, it has recently been described that persistent cell-cell contact can also down-modulate NKG2D expression. The goal of this study was to determine whether the NKG2D receptor is down-modulated by cell-cell contact with cervical cancer cells and whether this down-modulation might be associated with changes in NK cell activity.

**Results:**

We demonstrate that NKG2D expressed on NKL cells is down-modulated by direct cell contact with cervical cancer cell lines HeLa, SiHa, and C33A, but not with non-tumorigenic keratinocytes (HaCaT). Moreover, this down-modulation had functional implications. We found expression of NKG2D-ligands in all cervical cancer cell lines, but the patterns of ligand distribution were different in each cell line. Cervical cancer cell lines co-cultured with NKL cells or fresh NK cells induced a marked diminution of NKG2D expression on NKL cells. Additionally, the cytotoxic activity of NKL cells against K562 targets was compromised after co-culture with HeLa and SiHa cells, while co-culture with C33A increased the cytotoxic activity of the NKL cells.

**Conclusions:**

Our results suggest that differential expression of NKG2D-ligands in cervical cancer cell lines might be associated with the down-modulation of NKG2D, as well as with changes in the cytotoxic activity of NKL cells after cell-cell contact with the tumor cells.

## Background

Cervical cancer represents the third most commonly diagnosed cancer and the fourth leading cause of cancer-related deaths in women worldwide [1]. Human papillomavirus (HPV) infection is the most important risk factor for cervical cancer development [2,3]; persistence of high-risk HPV infection leads to premalignant lesions and may ultimately lead to cervical cancer in a multistep process [4,5]. The first line of defense against HPV in early infection is the innate immune system, which plays a crucial role in viral clearance [6].

Natural killer (NK) cells are an important arm of the innate immune system directly involved in the spontaneous recognition and lysis of virus-infected and tumor cells. NK cells are endowed with potent cytotoxic activity, and they can also produce several cytokines, such as IFN-γ, TNF-α, GM-CSF, IL-5, and IL-8 [7-10]. Evidence is also accumulating for the crucial role of NK cells in tumor immunosurveillance [11].

NK cell activity is finely regulated by an exquisite balance of inhibitory and activating receptors [12]. One of the best-characterized activating receptors is NKG2D, which recognizes two structurally distinct families of ligands. One of these is composed of MHC class I chain-related A and B (MICA/B) molecules. The other family of ligands is composed of the UL16-binding proteins (ULBPs) 1 to 6, originally identified through interactions with the human cytomegalovirus glycoprotein UL16 [13,14]. Both families are expressed on a wide range of epithelial tumors, such as colon carcinoma, breast cancer, neuroblastoma, and others [13,15-18].

It has been observed that some tumor cells are capable of releasing these regulating ligands. Tumor-derived soluble NKG2D-ligands have been shown to down-modulate the expression of NKG2D on NK cells and T cells, reducing their cytolytic activity [19]. Soluble MICA and soluble isoforms of ULBP2 and ULBP4 have been shown to bind to NKG2D leading to internalization of the receptor, which favors the development of NK cell-acquired dysfunction; soluble MICB has been shown to bind competitively to NKG2D and to inhibit the binding of NKG2D to transmembranal ligands expressed by tumor cells, thus blocking NK cell activation [19-21].

Recently, it has been shown that not only soluble forms of ligands are capable of inducing down-modulation of the NKG2D receptor. Some membrane-bound NKG2D-ligands, which are secreted via exosomes (such as MICA and ULBP3), are capable of down-modulating NKG2D expression on NK cells as well [22,23]. In recent years, some authors have demonstrated that NKG2D-ligands, by persistent cell-cell contact, are also able to cause the down-modulation of NKG2D receptors; however, the mechanism by which this phenomenon occurs is not yet clearly known [24-27].

The participation of NKG2D is known to occur in different tumors, such as melanoma, and ovarian, prostate, and colon carcinomas [26,28-30]. Unfortunately, despite the high frequency and mortality of cervical cancer around the world, the participation of the NKG2D receptor in either tumor progression or tumor elimination in cervical cancer is not yet fully understood. In this study, we investigated the expression of ULBP family proteins (MIC family expression has been already described in a previous report) in cervical cancer cell lines and the possible modulation of the NKG2D receptor in a well-established NK cell line (NKL), as well as in fresh NK cells. Likewise, the biological implications of NKG2D/NKG2D-ligand interaction on NK cell cytotoxic activity were also evaluated.

## Methods

### Cell cultures and reagents

Cervical cancer-derived cell lines (HeLa, SiHa, and C33A) and the spontaneously immortalized, but non-tumorigenic human epithelial cell line HaCaT (kindly obtained from Dr. P. Boukamp, of the German Cancer Research Center, DKFZ, Germany), were maintained in Dulbecco's modified Eagle's medium containing 2 mM L-glutamine supplemented with 10% fetal bovine serum, penicillin (100 U/mL) and streptomycin (100 μg/mL) (GIBCO™ Invitrogen Corporation, Carlsbad, CA, USA). The human NK cell line, NKL (kindly donated by Dr. M. Robertson of the Indiana University School of Medicine, IN, USA through Dr. M. Caligiuri of the Ohio State University, OH, USA) was used to monitor functional changes after exposing to cervical cancer cells. NKL cells were cultured in RPMI-1640 medium supplemented with 10% fetal bovine serum, penicillin (100 U/mL), streptomycin (100 μg/mL), and 200 U/mL of human recombinant IL-2 (Biolegend, San Diego, CA, USA). This medium has been referred as complete medium (CM). The human erythromyeloblastoid leukemia cell line K562 transfected with green fluorescent protein (GFP) was also cultured in RPMI-1640 medium. Cell cultures were maintained at 37°C in a humidified atmosphere containing 5% CO_2_.

### Evaluation of the ULBP family on cervical cancer cell surfaces

Cell surface expression of different ULBP family proteins (ULBP1-4) was detected by indirect staining protocol using a PE-conjugated anti-mouse secondary antibody after incubation with mouse anti-ULBP1-4 primary antibodies (Santa Cruz Biotech, Santa Cruz, CA, USA). At least 20,000 events were acquired per experiment using a FACSAria flow cytometer (BD Biosciences).

### Changes in NKG2D expression on NKL cells

In order to assess whether NKG2D expression was impaired after the contact with tumor cells, we co-cultured NKL cells with each cervical cancer cell line, as well as with the non-tumorigenic HaCaT cells. Each cell line was seeded in 6-well plates at a confluence of approximately 70%. Tumor or HaCaT cells were then co-cultured with NKL cells at E:T ratio of 1:1 for 1, 2, and 4 h at 37°C. Changes in NKG2D expression, given as mean fluorescence intensity (MFI), were monitored by flow cytometry using a PE-conjugated anti-human NKG2D monoclonal antibody (clone 1D11; Biolegend, San Diego, CA, USA). At least 20,000 events were acquired per cell line using a FACSAria flow cytometer (BD Biosciences). Experiments were performed in triplicate and repeated three times, for a total of nine data points per sample. Data are expressed as means ± SEM. The variations in the expression of NKG2D were expressed as percentages of increase or decrease with 100% defined as the basal NKG2D expression of NKL cells cultured alone (control group).

Additionally, we also evaluated changes in NKG2D expression on fresh NK cells after contact with cervical cancer cells or HaCaT keratinocytes. Briefly, fresh PBMC from healthy subjects were obtained on Ficoll-Hypaque gradients. After isolation, PBMC were adjusted at 1 × 10^6 ^cells/mL and incubated with anti-CD3-PECy7, anti-CD56-APC and anti-NKG2D-PE monoclonal antibodies. Changes in NKG2D expression were determined as described earlier.

### *In vitro *NKL cytotoxicity assays

To evaluate whether cytotoxic activity of NKL cells was affected by exposure to cervical cancer cell lines, we co-cultured NKL cells with the different cervical cancer cell lines, as well as with the non-tumorigenic HaCaT cells. HeLa, SiHa, C33A, and HaCaT cells were seeded in 6-well plates at a confluence of around 70% and then were placed in contact with NKL cells at E:T ratio of 1:1 for 4 h at 37°C. Then, the same NKL cells that had been co-cultured (or not, in the case of the control group) with the different cervical cancercell lines were challenged with GFP transfected-K562 cells in a GFP/propidium iodide (PI) flow cytometric assay [31]. Where indicated, anti-NKG2D monoclonal antibody (clone 1D11; Biolegend, San Diego, CA, USA) at blocking concentration of 30 μg/mL [32] was added to the NKL cells prior to the cytotoxicity assays.

NKL cells and target cells were incubated at different E:T ratios (1:1, 5:1, and 10:1) in complete medium for 3 h in a 5% CO_2 _atmosphere at 37°C. Cells were washed twice in PBS-1% BSA containing 0.1% sodium azide and incubated in the same buffer plus 20 μL/mL of PI for 15 min at 4°C in the dark. GFP-transfected K562 cells were incubated alone to measure basal cell death. At least 10,000 events were acquired for each sample passed through flow cytometer. Experiments for NKL cytotoxic activity were performed in triplicate and repeated three times, for a total of nine data points per sample. Data are expressed as means ± SEM. Analysis of data was performed using the WinMDI 2.9 software. Cytotoxic activity was expressed as a percentage of specific lysis and calculated by the following formula:

$$\%\text{ specific lysis}=\frac{\text{1}00(\%\text{ sample lysis}-\%\text{ basal lysis})}{\text{1}00-\%\text{ basal lysis}}$$

### Statistical analysis

Statistical analysis was performed using the GraphPad Prims 5 software (**GraphPad Software**, La Jolla, CA). Data were expressed as percentage and mean median fluorescence intensity (MFI). Significance was tested by using one-sample *t*-Test (theorical mean = 100%) for NKG2D downmodulation analysis and unpaired *t*-Test for cytotoxicity assays. Differences were considered statistically significant when the *p *value was < 0.05.

## Results

### NKG2D-ligands are differentially expressed by cervical cancer cell lines and non-tumorigenic keratinocytes

**Figure 1:**
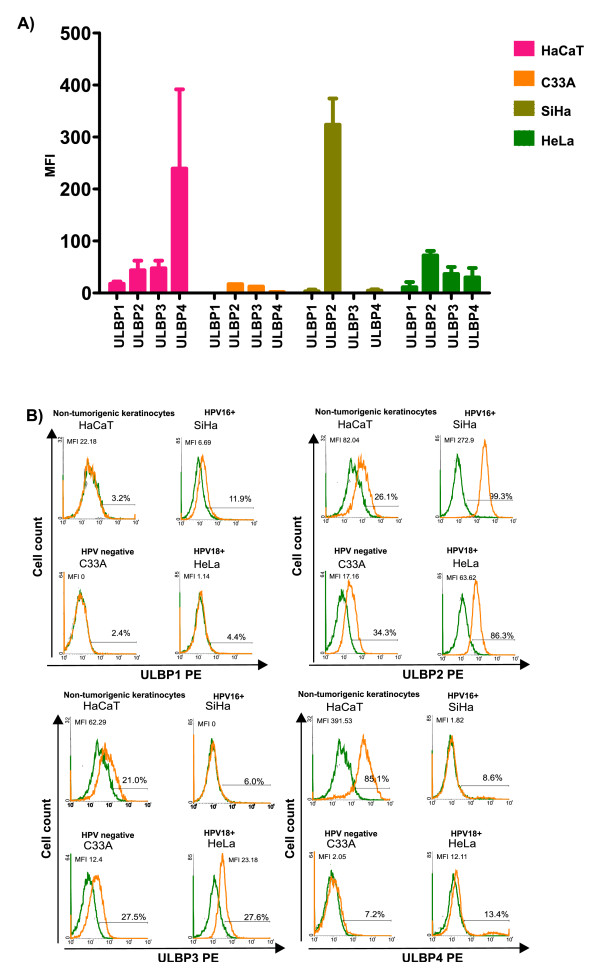
**Expression pattern of ULBP1-4 in different cervical cancer cell lines**. MFI of the different ULBP proteins in cervical cancer cell lines and non-tumorigenic HaCaT keratinocytes was measured by flow cytometry using a mouse anti-ULBP1, ULBP2, ULBP3, and ULBP4 primary antibodies followed by incubation with PE-conjugated anti-mouse secondary antibody **(A)**. Representative flow cytometry histograms for each cell line are shown in **(B)**. While ULBP1 is practically absent in all cell lines, ULBP2 is expressed universally, with cell surface staining especially high in SiHa and HeLa cells. ULBP3 expression is moderate in HaCaT, C33A, and HeLa cells, but absent in SiHa cells. ULBP4 expression is seen solely in the non-tumorigenic HaCaT keratinocytes..

Depending on their origin, cancer cell lines may express different levels of NKG2D-ligands [33]. In this study, we investigated the expression of ULBP1-4 ligands in cervical cancer cell lines infected with HPV16 (SiHa), HPV18 (HeLa), HPV-negative (C33A), and non-tumorigenic keratinocytes (HaCaT). While ULBP1 expression was practically absent in all cell lines, ULBP2 was observed in all cell lines, being especially high in SiHa and HeLa cells (Figure 1). ULBP3 was present in HeLa, C33A, and HaCaT. Conversely, SiHa was totally negative for ULBP3 (Figure 1). Interestingly, ULBP4 expression was only seen in HaCaT cells (immortalized, although non-tumorigenic keratinocytes), as also shown in Figure 1. Additionally, in this study we corroborated our previous report that MICA expression was higher than MICB expression in HeLa and SiHa cells. In contrast, MICB was higher in C33A cells (data not shown) as we had likewise demonstrated in a previous report [34].

### Cervical cancer cell lines down-modulate NKG2D expression on NKL cells

**Figure 2:**
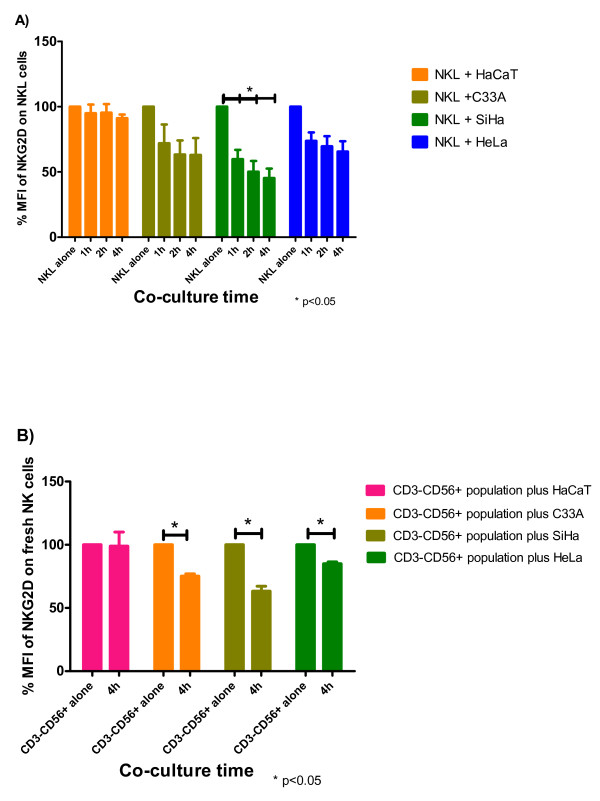
**Down-modulation of NKG2D on NKL cells and fresh NK cells after cellcell contact with cervical cancer cells.** NKG2D expression was evaluated by flow cytometry using a PE-conjugated anti-human NKG2D monoclonal antibody. The variations in the MFI of NKG2D are expressed as percentages with 100% defined as the basal MFI of NKG2D on NKL cells or fresh NK cells cultured alone (control group). **(A)** NKL cells co-cultured with HaCaT cells do not show significant changes in the expression of NKG2D after 60 min; this behavior remains practically the same after 2 and 4 h. In contrast, the co-culture with cervical cancer cells results in NKG2D downmodulation. After incubation with SiHa (HPV16+) and HeLa (HPV18+) cells the NKG2D expression is significantly reduced after 60 min and continues to decrease over time. The co-cultures of NKL cells with C33A cells (which are negative for HPV) show a similar pattern to that observed with SiHa and HeLa. Notably, the strongest decrease in NKG2D expression is observed after incubating the NKL cells with SiHa cells. Experiments were performed in triplicate and repeated three times, for a total of nine data points per sample. Data are expressed as mean ± SEM. *p < 0.05. **(B)** Fresh NK cells significantly down-modulated NKG2D after the contact with SiHa, HeLa and C33A cells; conversely, after the contact with HaCaT cells, the expression of NKG2D was not modified. Experiments were performed in duplicate and repeated two times with two different donors. Data are expressed as mean ± SEM. *p < 0.05.

Some tumors and cancer cell lines are able to modulate the expression of NKG2D on NK cells and T lymphocytes after cell-cell contact; this modulation has been shown to have functional effects [27]. In this study, we investigated the possible down-modulation of NKG2D on NKL cells after cell contact with cervical cancer cell lines and non-tumorigenic HaCaT keratinocytes. NKL cells were co-cultured with cervical cancer cells and changes in the expression of NKG2D were evaluated at different time points. When co-cultured with HaCaT cells, no significant changes in the expression of NKG2D were observed after 60 min (only 5.02% reduction); this behavior remained practically the same after 2 and 4 h. In contrast, the co-culture of NKL cells with cervical cancer cells resulted in NKG2D down-modulation. After incubation with C33A cells (which are negative for HPV), the expression of NKG2D was reduced after 60 min (42.3% reduction) and continued to decrease over the next two time points (42.3% and 49.9% reduction at 2 and 4 h, respectively). The co-culture of NKL cells with SiHa (HPV16+) also showed a strong decrease in the expression of NKG2D with a diminution of 47.4% during the first 60 min. NKG2D expression continued to decrease over time (57.9% and 61.7% reduction after 2 and 4 h, respectively). HeLa (HPV18+) showed similar behavior, although the effect was less than that observed with SiHa and C33A (27.4%, 29.4% and 35.2% reduction after 1, 2, and 4 h, respectively). Notably, the strongest decrease in NKG2D expression was observed after incubating the NKL cells with SiHa cells (Figure 2A).

Reinforcing our results with the NKL cells, we also observed a significant down-modulation of NKG2D on fresh NK cells after the contact with tumor cells. In contrast, after contact with HaCaT cells, the expression of NKG2D remained practically the same (Figure 2B).

### Co-culture of NKL cells with different cervical cancer cell lines modulates cytotoxic activity of NKL cells

**Figure 3:**
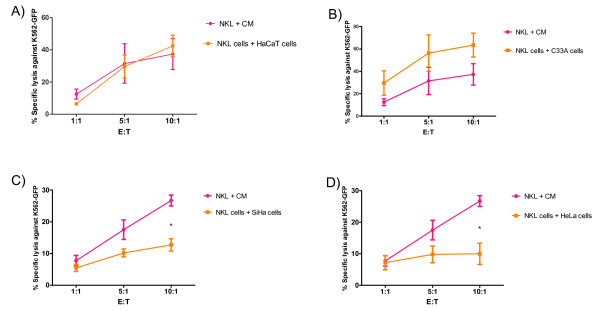
**NKL cell cytotoxic activity is impaired after exposing NKL cells with cervical cancer cells**. NKL cells were co-cultured with the different cell lines for 4 h. Subsequently, the NKL cells were removed from each co-culturing cells (SiHa, HeLa, C33A or HaCaT) and the cytotoxicity was evaluated. The NKL cells were incubated for 3 h using GFP-transfected K562 as target cells at different E:T ratios (1:1, 5:1, and 10:1) for 3 h. Dead target cells were discriminated based on GFP + and PI + population. The co-culture of NKL with HaCaT cells does not produce any significant change in NKL cytotoxic activity (**A**). After co-culture with C33A, we see an increase in the cytotoxic activity of NKL cells (**B**). In contrast, co-cultures with SiHa (**C**) and HeLa cells (**D**) considerably decrease the cytotoxic activity of NKL cells. Experiments were performed in triplicate and repeated three times, for a total of nine data points per sample. Data are expressed as mean ± SEM. * *p *< 0.05, CM: complete medium.

**Figure 4:**
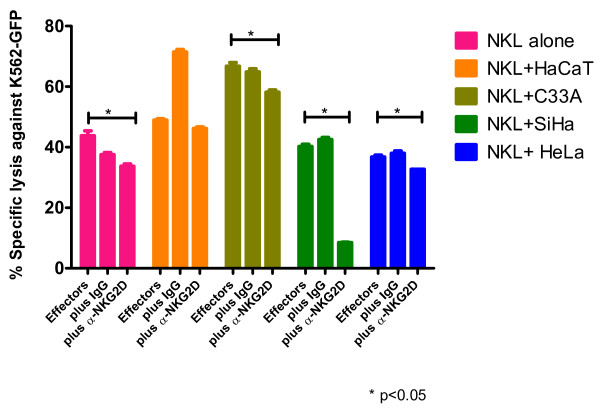
**Blocking of the NKG2D receptor on effector NKL cells**. NKL cells were co-cultured with the different cell lines for 4 h. The NKL cells were subsequently removed from co-culturing cells (SiHa, HeLa, C33A or HaCaT) and the NKL cells were additionally incubated with an anti-NKG2D monoclonal antibody at blocking concentration (30 μg/mL) or with IgG1 isotype control (clone MOPC-21; Biolegend, San Diego, CA, USA). Afterwards, NKL cells were incubated for 3 h using GFP-transfected K562 as target cells at an E:T ratio of 10:1. Dead target cells were discriminated based on GFP + and PI + population. The addition of blocking anti-NKG2D monoclonal antibody to the NKL cells had an additive effect on the previoulsy observed cytotoxicity diminution due to co-culture; this effect was dramatically more evident on NKL cells previously exposed to SiHa cells. Experiments were performed in duplicate and repeated four times. Data are expressed as mean ± SEM. * *p *< 0.05.

Next, we determined whether NKG2D down-modulation had any effect on the cytotoxic activity of NKL cells. NKL cells were co-cultured with several cervical cancer cell lines for 4 h. Following this, NKL cells were removed and incubated with green fluorescent protein (GFP)-transfected K562 cells for cytotoxicity assays using flow cytometry. Gating of the dead tumor cells was based on the GFP and propidium iodide (PI) double positive population. The percentage of specific lysis was calculated as described in the "Methods" section. When NKL cells were co-cultured with HaCaT cells, no changes in the cytotoxic activity were observed (Figure 3). In contrast, co-cultures with SiHa and HeLa cells, both HPV-positive, showed a significant reduction in the NKL cytotoxic activity (Figure 3). Unexpectedly, NKL cells co-cultured with C33A cells (HPV-negative) showed increased cytotoxicity against K562 cells when compared with the control, non-co-cultured cells (Figure 3). As there is strong evidence that NK cells participate directly in anti-tumor immune responses through NKG2D engagement, we designed blocking experiments using an anti-NKG2D monoclonal antibody at 30 μg/mL. Results showed that NKG2D blockage had an additive effect on the cytotoxicity diminution. This effect was more evident when NKL cells were previously incubated with SiHa cells (Figure 4).

## Discussion

It is well accepted that NK cells play a key role in the innate immune system's surveillance against tumors [35,36]. Tumor surveillance requires the interaction between NK cell triggering receptors and their ligands. NK cells express major histocompatibility complex (MHC) class I-specific receptors, such as killer cell immunoglobulin-like receptors (KIRs), which may activate or inhibit NK-cell effector functions [37]. Inhibitory KIR receptors allow NK cells to recognize cells that express self-MHC-I molecules and subsequently inhibit NK cell effector functions [38]. With the loss of these inhibitory interactions; however, target cells become susceptible to NK cell activity via the triggering of NK cell-activating receptors [39,40]. One of these receptors is NKG2D, which recognizes two different groups of MHC-I-like molecules, the MIC and the ULBP/RAET1 families. These ligands have been found to be expressed in a wide range of different solid tumors and in some normal tissues [15-18,41]. The recognition of MICs or ULBPs by NKG2D induces NK cell activation; however, recent studies have shown that NKG2D-ligands are also capable of inducing the down-modulation of NKG2D, and subsequent loss of NK activation, via persistent cell contact between NKG2D and NKG2D-ligands [42].

In this study we have shown that cervical cancer cell lines differentially express both families of NKG2D-ligands. That is, MICA (data not shown) and ULBP2 are preferentially expressed by HPV-positive cell lines (HeLa and SiHa), whereas the HPV-negative cell line, C33A, preferentially expresses MICB (data not shown). Interestingly, ULBP4 was only expressed by the non-tumorigenic HaCaT keratinocytes. This differential expression of the NKG2D-ligands raises the question of whether HPV infection could play a role in their regulation. Several studies have shown that some viruses, such as human cytomegalovirus and herpesvirus, are capable of modulating the expression of NKG2D-ligands as an immune evasion mechanism. Both viruses produce immunoevasins that target the NKG2D-ligands MICA, MICB, and ULBP1-3. The intracellular retention of these ligands inhibits their cell surface expression [43-45]. At this time, it is unknown whether HPV possesses similar mechanisms for the evasion of NKG2D-mediated NK responses.

The oncoproteins E6 and E7 of high-risk HPV are capable of interfering with the binding of certain immunoproteins, such as transcriptional factors (IRF3, IRF1) involved in innate immune responses [46-48]. A recent study showed that the HPV16 E5 oncoprotein intracellularly binds the CD1 molecule and inhibits its expression in a HPV16 E5-transfected cervical cancer cell line [49]. This finding raises the question as to whether HPV-derived proteins might also be capable of binding other MHC class I-like molecules, such as NKG2D-ligands. Therefore, it will be interesting to design co-localization experiments to determine if any of the HPV oncoproteins could also intracellularly sequester NKG2D-ligands, leading to functional NK cell and T cell defects.

It has been previously demonstrated that several tumors expressing NKG2D-ligands induce the down-modulation of NKG2D on NK cells and T cells. In the present study, we co-cultured different cervical cancer cell lines and non-tumorigenic HaCaT keratinocytes with an NK cell line, NKL, and observed a very strong down-modulation of NKG2D expression after contact with all cervical cancer cell lines (SiHa, HeLa, and C33A). Like NKL cells, fresh NK cells were also susceptible to down-modulate NKG2D after the contact with tumor cells. This reduction in cell surface expression of NKG2D is similar to recent reports showing that this phenomenon can be induced by other tumor cells [27].

The down-modulation of NKG2D, by binding to either soluble or transmembranal forms, has been shown to have functional implications for NK cells and T cells, leading to impaired NK cell activity and poor cytotoxicity [21,42]. In this study, we evaluated whether the negative regulation of NKG2D expression on NKL cells had functional effects on the cytotoxic activity of these cells. We found that NKL cell cytotoxic activity against target cells (K562) diminished after contact with the HPV-positive cervical cancer cell lines, SiHa and HeLa, but not after contact with HPV-negative C33A cells and non-tumorigenic HaCaT keratinocytes. Due to the fact that C33A cells caused NKG2D down-modulation, we would have expected to find impairments in NK cell activity. However, co-culture of NKL cells with C33A cells led to increased cytotoxic activity against K562 cells when compared against both the control and HPV-infected cells. At this point, it is important to note that C33A cells expressed a different pattern of NKG2D-ligands than the other cell lines. Notably, MICA (data not shown) and ULBP2 were lower than SiHa and HeLa cells, while MICB levels were higher in C33A cells (data not shown). A recent study showed that transmembranal MICB is capable of inducing NK cell activation via the NKG2D receptor and that this ligand does not participate in negative modulation of NK cell activity [29]. Thus, it is logical to speculate that the differences in the patterns of NKG2D-ligand expression, specifically the higher levels of MICB, could be responsible for changes in the cytotoxicity of the NKL cells against the K562 target cells. However, we cannot disregard the possible role of soluble ligand expression. We have recently found that soluble MICB is also greatly increased in C33A cells compared with SiHa and HeLa [34].

One important further experiment will be to knock-down NKG2D expression in NKL cells and to verify whether the loss of this activating receptor explains the loss of NKL-mediated cytotoxicity against K562 targets. This experiment will be important in order to clarify whether other interactions between the tumor cells and NKL cells might also be responsible for the loss of cytotoxicity observed in our experimental setting.

In order to clarify the role of NKG2D in NKL cytotoxic activity, we performed experiments using a blocking anti-NKG2D monoclonal antibody. Intriguingly, the addition of the blocking antibody to NKL cells previously exposed to SiHa cells resulted in a significant diminution of the previously observed cytotoxicity. This could be due to the fact that this cell line is unique (among the cell lines in this study) in its strong expression of the NKG2D ligand ULBP2. While the antibody blockade of NKG2D also significantly inhibited cytotoxicity in unexposed and in NKL cells previously exposed to the other cancer cell lines, this result was not nearly as marked as the result seen with SiHa pre-exposure. Certainly, this inhibition was only partial, which indicates that other activating receptors, such as NKp30 or NKp46 (moderately expressed by our NKL cells) might also be contributing to the tumor cell lysis or that the numbers of anti-NKG2D antibody (at the blocking concentrations used) were not sufficient to block all the receptors remaining on the cell membrane. In summary, it will be important to conduct experiments with blocking antibodies against other important activating receptors, including NKp30, NKp46, and DNAM-1, which might lead to tumor cell-NK cell interactions as well.

## Conclusions

Taken together, our results suggest that cervical cancer cell lines expressing NKG2D-ligands induce immunoregulation in NKL cells via down-modulation of NKG2D. This down-modulation leads to a reduction of the NKL cell cytotoxic activity against K562 target cells. Thus, these results indicate that the complexity of tumor immunosuppressive mechanisms should be considered before designing NK cell-based therapies in cervical cancer patients.

## Abbreviations

HPV: Human Papilloma Virus; KIR: Killer cell immunoglobulin-like receptor; MHC-I: Major Histocompatibility Complex class I; MICA: MHC class I-related chain A; MICB: MHC class I-related chain B; NKG2D: Natural-killer group 2 member D; ULBP: UL-16 Binding Protein.

## Competing interests

The authors declare that they have no competing interests.

## Authors' contributions

MIJP performed the experimental work, searched scientific literature and contributed to the draft of the manuscript. LFJS and PCOL contributed to the theoretical framework of the study and provided scientific guidance throughout the project. ABC, GHF and ADN contributed to the planning of the project and provided valuable scientific suggestions. AAL provided scientific guidance throughout the project and participated with experimental work. OGR and ALPS contributed to the draft of the manuscript and provided valuable scientific suggestions. STA conceived and designed the theoretical framework of the study, provided scientific guidance throughout the project and wrote the manuscript. All authors read and approved the final manuscript.
